# Oxytocin Differentially Modulates Amygdala Responses during Top‐Down and Bottom‐Up Aversive Anticipation

**DOI:** 10.1002/advs.202001077

**Published:** 2020-07-01

**Authors:** Fei Xin, Xinqi Zhou, Debo Dong, Zhongbo Zhao, Xi Yang, Qianqian Wang, Yan Gu, Keith M. Kendrick, Antao Chen, Benjamin Becker

**Affiliations:** ^1^ The Clinical Hospital of Chengdu Brain Science Institute MOE Key Laboratory for Neuroinformation University of Electronic Science and Technology of China Xiyuan Avenue 2006 Chengdu 611731 China; ^2^ Key Laboratory of Cognition and Personality Ministry of Education, Faculty of Psychology Southwest University Tiansheng Road 2 Chongqing 400715 China

**Keywords:** amygdala, anticipation, anxiety, functional magnetic resonance imaging (fMRI), oxytocin

## Abstract

The ability to successfully regulate negative emotions such as fear and anxiety is vital for mental health. Intranasal administration of the neuropeptide oxytocin (OXT) has been shown to reduce amygdala activity but to increase amygdala–prefrontal cortex connectivity during exposure to threatening stimuli suggesting that it may act as an important modulator of emotion regulation. The present randomized, between‐subject, placebo‐controlled pharmacological study combines the intranasal administration of OXT with functional magnetic resonance imaging (fMRI) during an explicit emotion regulation paradigm in 65 healthy male participants to investigate the modulatory effects of OXT on both bottom‐up and top‐down emotion regulation. OXT attenuates the activation in the posterior insular cortex and amygdala during anticipation of top‐down regulation of predictable threat stimuli in participants with high trait anxiety. In contrast, OXT enhances amygdala activity during the bottom‐up anticipation of unpredictable threat stimuli in participants with low trait anxiety. OXT may facilitate top‐down goal‐directed attention by attenuating amygdala activity in high anxiety individuals, while promoting bottom‐up attention/vigilance to unexpected threats by enhancing amygdala activity in low anxiety individuals. OXT may thus have the potential to promote an adaptive balance between bottom‐up and top‐down attention systems depending on an individual's trait anxiety level.

## Graphical abstract

The present study provides direct evidence that intranasal treatment of oxytocin attenuates bilateral posterior insular cortex and right amygdala activation during anticipation of top‐down regulation of predictable threat stimuli in participants with high trait anxiety, whereas oxytocin increases activation in the amygdala during anticipation of bottom‐up unpredictable threat stimuli in participants with low trait anxiety.

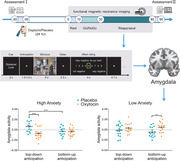

## 1 Introduction

From an evolutionary perspective, fear and anxiety elicit defensive responses to detect, react, and cope with the imminent or potential threat and to avoid harm.^[^ ^1^ ^]^ Both excessive and deficient anxiety and fear strongly interfere with the ability to react to and cope with the potential threat and challenging life situations, as exhibited for instance in anxiety disorders or as a result of brain lesions.^[^ ^2^, ^3^ ^]^

The ability to successfully regulate negative emotions such as fear and anxiety is vital for mental health, well‐being, and social functioning.^[^ ^4^, ^5^ ^]^ Consequently, dysregulations in the fine‐grained interplay between the generation and regulation of negative emotions represent transdiagnostic deficits across major psychiatric disorders.^[^ ^6^, ^7^ ^]^ Behavioral interventions,^[^ ^8^, ^9^, ^10^ ^]^ real‐time functional magnetic resonance imaging (fMRI) neurofeedback guided modulation of amygdala‐prefrontal circuits,^[^ ^11^, ^12^ ^]^ and pharmacological agents primarily targeting serotonergic and GABA‐ergic neurotransmission,^[^ ^13^, ^14^ ^]^ can facilitate the regulation of negative emotions, including anxiety and fear. However, not all patients respond adequately to the currently available treatment options and the pharmacological interventions may induce negative side effects which often limit compliance with the treatment protocols. Therefore, novel strategies to improve emotion regulation are urgently needed.

Emotion generation and regulation involve the interaction of intrinsic/automatic bottom‐up processes and controlled/deliberate top‐down processes.^[^ ^15^, ^16^, ^17^ ^]^ Bottom‐up processes are stimulus‐driven and initiated by salient stimuli in the environment. In contrast, top‐down processes are goal‐driven and context‐dependent.^[^ ^18^, ^19^, ^20^ ^]^ During both the (pre‐stimulus) anticipation and the advent of the aversive events, the bottom‐up and top‐down processes rely on differential neural systems and temporal dynamics. Bottom‐up emotional processes are mediated by subcortical systems such as the amygdala and hypothalamus. In contrast, top‐down regulatory processes rely on prefrontal executive control systems.^[^ ^21^, ^22^ ^]^ Relative to automatic stimulus‐driven bottom‐up processes, top‐down processes take into account the context of threat (predictive) cues and initiate anticipatory goal‐directed responses to facilitate threat‐vigilance and emotion regulation. Although anticipatory cues or goals can induce higher levels of anxiety, they may also facilitate perceptual and attentional prioritization of threatening stimuli such that anticipatory cues can accelerate stimulus coding in visual and attention systems.^[^ ^20^, ^23^, ^24^, ^25^ ^]^

The anticipatory response to low imminent, uncertain, or future threat specifically refers to anxiety whereas fear is elicited by high imminent and certain threat.^[^ ^26^, ^27^, ^28^ ^]^ The neural circuits that mediate fear‐associated responses and the implicit and explicit regulation of imminent threat,^[^ ^29^ ^]^ as well as dysregulations in these processes in psychiatric disorders,^[^ ^6^ ^]^ have been extensively studied. However, despite the important role of exaggerated threat anticipation and impaired regulation of the anticipatory response for the pathophysiological mechanism of anxiety disorders,^[^ ^30^, ^31^ ^]^ only a few studies focused on the pre‐stimulus anticipatory period. Previous studies indicate that—in accordance with the proposed pathological mechanism in anxiety disorders—pre‐stimulus anticipatory brain activity can predict subsequent emotion regulation success or failure.^[^ ^32^ ^]^

Previous animal models and human studies revealed extensive evidence for an important role of the hypothalamic neuropeptide oxytocin (OXT) in anxiety. Based on these findings intranasal OXT has been increasingly suggested as a potential promising pharmacological treatment for anxiety and fear‐related disorders.^[^ ^33^, ^34^, ^35^, ^36^ ^]^ Converging evidence indicates an anxiolytic potential of intranasal OXT reflected by reduced amygdala activity and increased amygdala–prefrontal cortex (PFC) functional connectivity in response to threatening stimuli in healthy adults.^[^ ^34^, ^37^, ^38^, ^39^ ^]^ Two of the key regions modulated by OXT, amygdala, and PFC, play crucial roles in emotion generation and regulation.^[^ ^29^, ^40^, ^41^ ^]^ In addition to effects on this circuitry in response to imminent threat stimuli, intranasal OXT has been repeatedly demonstrated to enhance intrinsic amygdala–prefrontal connectivity in the absence of threat‐related stimuli, suggesting potential effects of OXT on the anxiety‐related circuitry that precede the actual encounter of threat.^[^ ^42^, ^43^ ^]^ The effects of OXT on human cognition and behavior are partly mediated through widely distributed central OXT receptors, with a particularly dense expression of OXT‐sensitive receptors in the neural systems associated with emotion generation and regulation, including the prefrontal and anterior cingulate cortex, as well as the amygdala, hippocampus, striatum, and the brainstem.^[^ ^44^, ^45^, ^46^ ^]^ Based on these findings, we expected that OXT may modulate anticipatory amygdala threat responses and the regulation of anticipatory threat responses in the PFC.

**Figure 1:**
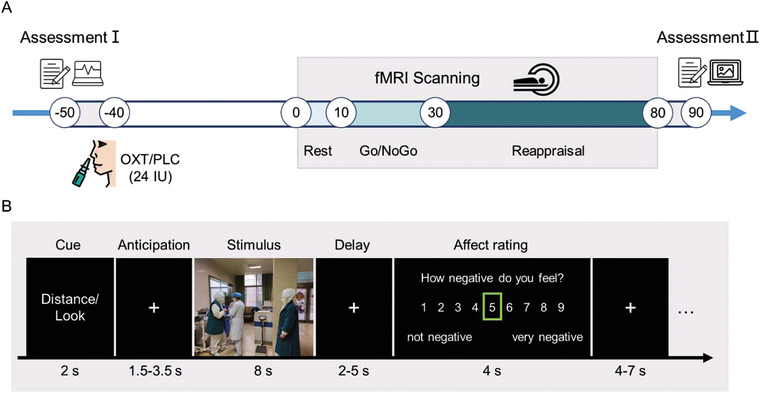
A) Experimental protocols. B) Schematic representation and timing of the reappraisal paradigm. For copyright reasons, the picture shown as an example is not from the IAPS, and permission was obtained.

To investigate the modulatory effects of OXT on both bottom‐up and top‐down emotion regulation, we conducted a randomized, between‐subject, placebo (PLC)‐controlled pharmacological fMRI study that combined the intranasal administration of OXT with an explicit emotion regulation paradigm (cognitive reappraisal) in 65 healthy male participants (see **Figure** **1**A for a schematic depiction of the experimental protocols). Cognitive reappraisal represents a commonly employed strategy for regulating emotional reactivity to aversive stimuli and refers to the explicit modulation of the self‐relevant meaning of an emotion‐inducing stimulus.^[^ ^17^, ^29^ ^]^ The cognitive reappraisal paradigm employed in the present study (see also schematic depiction in Figure 1B) includes i) a “Look” condition during which participants are instructed to simply view threatening or neutral images and react naturally (bottom‐up trials) and ii) a “Distance” condition during which participants are instructed to view threatening images and downregulate their negative emotional response by means of distancing as a cognitive reappraisal strategy (top‐down trials).^[^ ^47^, ^48^, ^49^ ^]^ In accordance with previous studies, an index of reappraisal success was defined as the mean decrease of the negative affect reported during reappraisal (“Distance”) trials relative to that reported on emotional reactivity (“Look”) trials in response to highly negative stimuli.^[^ ^50^ ^]^ Larger difference scores thus correspond to a greater reduction in negative affect and reflect higher reappraisal success. The paradigm additionally includes a pre‐stimulus anticipatory period between a cue signaling the instruction for the subsequent stimulus (“Look” or “Distance”), which allows examination of anticipatory responses to uncertain or certain subsequent threat, given that the “Look” cue is followed in 50% of the trials by a threatening or neutral stimulus but the “Distance” cue is followed in 100% of the trials by a threatening stimulus. We primarily focused on the amygdala and PFC activation during the pre‐stimulus anticipation and stimulus presentation periods to examine the effects of OXT on anticipatory bottom‐up and top‐down emotion regulation.

Accumulating evidence indicates that the behavioral and neural effects of OXT may be moderated by individual differences in pre‐treatment trait anxiety and emotion regulation capability, such that OXT may produce a more pronounced anxiolytic effect in individuals with high trait anxiety^[^ ^51^, ^52^ ^]^ and low emotion regulation ability.^[^ ^53^ ^]^ Individuals with high trait anxiety additionally exhibit enhanced perceptual sensitivity to threat‐related stimuli as well as associated activation in amygdala‐frontal circuits.^[^ ^54^ ^]^ Based on these findings we additionally hypothesized that the effects of OXT and the susceptibility to threatening information are modulated by individual variations in pre‐treatment trait anxiety and emotion regulation ability.

## 2 Results

### 2.1 Demographics and Questionnaires

The OXT (*n* = 35) and PLC (*n* = 30) groups did not differ in age, levels of depression, mood, anxiety, autistic traits, and self‐control ability (detailed group characteristics are given in **Table** **1**).

**Table 1:** Demographics, depression, trait autism, anxiety levels and trait emotion regulation in the treatment groups receiving oxytocin (OXT) or placebo (PLC), respectively

	PLC (*n* = 30)	OXT (*n* = 35)	*T*	*p*
Age [years]	20.77 ± 3.05	20.89 ± 2.19	0.183	0.856
BDI‐II	6.23 ± 5.33	8.03 ± 6.07	1.257	0.213
AQ	21.53 ± 5.09	22.03 ± 4.62	0.411	0.682
ACS‐AOF	5.77 ± 3.21	6.03 ± 2.74	0.355	0.724
ACS‐AOD	6.73 ± 2.89	6.09 ± 2.67	−0.938	0.352
ACS‐AOP	8.80 ± 2.41	8.71 ± 1.89	−0.161	0.873
Pre‐treatment				
STAI‐state	36.67 ± 6.46	39.03 ± 6.97	1.409	0.164
STAI‐trait	38.17 ± 6.50	40.23 ± 7.33	1.191	0.238
PANAS positive	23.17 ± 6.54	23.49 ± 7.82	0.177	0.860
PANAS negative	14.70 ± 6.98	16.17 ± 6.87	0.855	0.396
Post‐scanning				
STAI‐state	36.07 ± 9.76	39.97 ± 10.12	1.576	0.120
STAI‐trait	37.53 ± 7.89	40.83 ± 9.29	1.527	0.132
PANAS positive	22.33 ± 6.34	19.89 ± 7.45	−1.413	0.162
PANAS negative	13.03 ± 6.57	14.23 ± 7.52	0.677	0.501

Data are expressed as mean ± SD (SD: standard deviation). For continuous variables, independent sample *t*‐tests were carried out.

### 2.2 Behavioral Results

**Figure 2:**
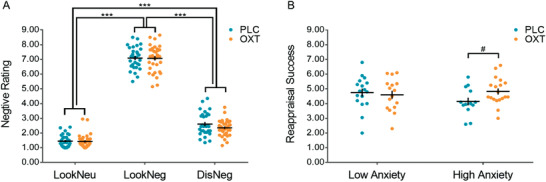
A) Behavioral performance (mean ± standard error) on the emotion regulation task. B) Oxytocin effects on reappraisal success (mean ± standard error) in high and low anxiety groups. 0.05 ≤ ^#^ *p* < 0.10; *p < 0.05; **p < 0.01; ***p < 0.001.

In post‐fMRI assessments, all participants reported that they successfully implemented the distancing strategy as instructed (extent of success: Mean ± SD = 5.66 ± 0.59, rating scale ranging from 1 for not successful at all to 7 for very successful). Examining the effects of OXT on self‐reported negative affect ratings during the fMRI paradigm by means of a mixed‐effect analysis of variance (ANOVA) with the between‐subject factors Treatment (OXT, PLC) and Anxiety (high, low trait anxiety) and the within‐subject factor Condition (Look Neutral, LookNeu; Look Negative, LookNeg; Distance Negative, DisNeg) revealed a significant main effect of Condition (*F*[2, 60] = 2300.48, *p* < 0.001, *η* ^2^ *_p_* = 0.987), but no main effect of Treatment (*p* = 0.505) or Anxiety (*p* = 0.260). We also did not observe a two‐way Treatment × Condition interaction (*p* = 0.420), a two‐way Treatment × Anxiety interaction (*p* = 0.614), or a three‐way interaction between the factors (*p* = 0.129). Post hoc tests revealed that: i) negative affect rating was significantly higher during the LookNeg condition than the LookNeu condition (*p* < 0.001), confirming successful induction of moderate negative affect; ii) during the reappraisal (DisNeg) condition significantly less negative affect was reported compared to the LookNeg condition (*p* < 0.001), confirming successful regulation of negative affect in the entire sample (**Figure** **2**A).

Based on our hypothesis we explored the interaction effect of treatment and trait anxiety on reappraisal success (i.e., reductions in reported negative affect, LookNeg – DisNeg, details see Section 5.5.) using a univariate ANOVA with the factors Treatment (OXT, PLC) and Anxiety (high, low). There were no significant main effects of Treatment (*p* = 0.306) and Anxiety (*p* = 0.459), nor a significant Treatment × Anxiety interaction effect (*p* = 0.108). Based on increasing evidence that OXT may produce more pronounced effects in individuals with high trait anxiety,^[^ ^51^, ^52^ ^]^ exploratory post hoc simple effects analyses within the anxiety groups were performed. Post hoc Bonferroni‐corrected analyses showed a marginally significant improvement in regulation success following OXT in the high anxiety group (reappraisal success: PLC = 4.14 ± 0.94, OXT = 4.82 ± 0.91, *p* = 0.069, see Figure 2B).

### 2.3 fMRI Results

#### 2.3.1 Top‐Down Guidance of Threat‐Related Perception and Attention

**Figure 3:**
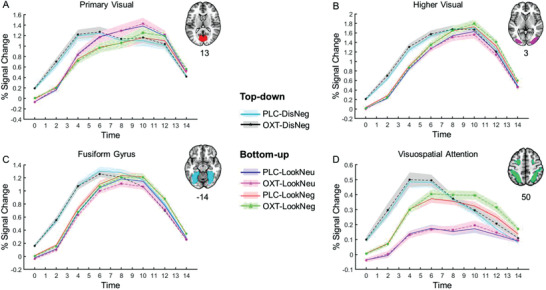
Top‐down cue (i.e., “Distance”) accelerates stimulus coding in the visual and attention systems. Time courses were extracted from atlas‐based regions in the A) primary visual network, B) higher visual network, C) fusiform gyrus, and D) visuospatial attention network.

In order to investigate the top‐down and bottom‐up properties of the emotion regulation paradigm, we initially extracted the time courses from the clusters in the primary visual network, higher visual network, fusiform gyrus, and visuospatial attention network during stimulus presentation. The time courses confirmed that the “Distance” cue accelerated the onset of stimulus coding, suggesting that the visual and attention networks were more rapidly engaged after top‐down “Distance” cues compared to bottom‐up “Look” cues (see **Figure** **3**).

#### 2.3.2 Oxytocin Differentially Modulated Amygdala Responses between High and Low Anxiety during Top‐Down (Distance) and Bottom‐Up (Look) Aversive Anticipation

**Figure 4:**
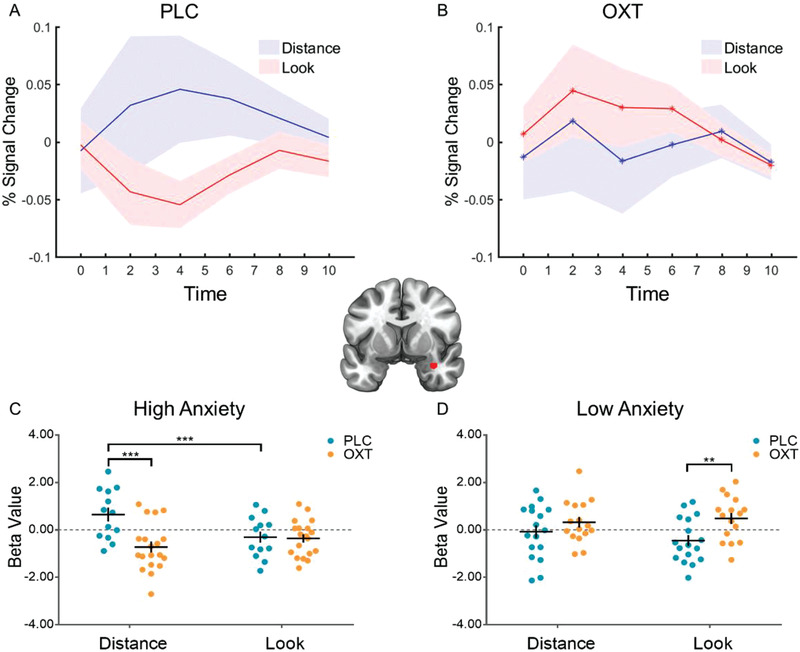
Oxytocin differentially modulated amygdala responses during distance and look threat anticipation. Time course of the right amygdala (red cluster) in the A) PLC and B) OXT groups. C) OXT attenuated anticipatory amygdala activity following the “Distance” cue in the high anxiety participants. D) OXT increased anticipatory amygdala activity following the “Look” cue in the low anxiety participants. *p < 0.05; **p < 0.01; ***p < 0.001.

The whole‐brain full factorial ANOVA model with the factors Treatment (OXT, PLC) and Anticipation (Look, Distance) revealed a significant Treatment × Anticipation interaction effect located in the bilateral posterior insular cortex (left posterior insula/parietal operculum: [−48, −36, 18], *p* = 0.001; right posterior insula/central operculum: [48, −9, 12], *p* = 0.047; cluster‐level family‐wise error (FWE) corrected) and the right amygdala (right: [33, 0, −24], *p* = 0.001; SV (small volume) FWE‐corrected, see Figure S3, Supporting Information). Post‐hoc examination of the extracted parameter estimates from the regions demonstrating significant interaction effects revealed that OXT significantly attenuated activity in the left posterior insula (*t*(63) = −3.384, *p* = 0.001) and the right posterior insula (*t*(63) = −4.200, *p* < 0.001) during distance anticipation in the OXT relative to the PLC group. Moreover, we observed that OXT differentially modulated amygdala activity for “Look” and “Distance” anticipation. Specifically, OXT marginally significantly attenuated amygdala activity during distance anticipation (*t*(63) = −1.747, *p* = 0.085), but marginally significantly enhanced amygdala activity during look anticipation (*t*(63) = 1.824, *p* = 0.073). Subsequent extraction of the time‐courses from the identified amygdala region (4‐mm radius sphere, centered at [33, 0, −24]) further demonstrated that OXT decreased amygdala activity during the anticipation of a certain threat (“Distance”), but increased it during uncertain threat anticipation (“Look”) (**Figure** **4**A,B).

Additional exploratory analyses were conducted to further elucidate the modulatory effect of trait anxiety on the neural effects of OXT. To this end parameter estimates from the amygdala were subjected to a three‐way mixed ANOVA with the factors Treatment (OXT, PLC), Anxiety (high, low), and Anticipation (Look, Distance). We observed a significant Treatment × Anxiety interaction effect (*F*[1, 61] = 10.840, *p* = 0.002, *η* ^2^ *_p_* = 0.151) and a marginally significant Treatment × Anxiety × Anticipation interaction effect (*F*[1, 61] = 2.904, *p* = 0.093, *η* ^2^ *_p_* = 0.045). Post hoc Bonferroni‐corrected analyses confirmed that the effects of OXT were modulated by differences in trait anxiety. Specifically, OXT significantly attenuated anticipatory amygdala activity in the high trait anxiety group (*p* = 0.021), but significantly increased anticipatory amygdala activity in the low trait anxiety group (*p* = 0.026). An exploratory post hoc three‐way interaction analysis on the extracted parameter estimates from this region furthermore confirmed that OXT significantly attenuated amygdala activity during distance anticipation (*p* < 0.001) in the high trait anxiety group (Figure 4C), but significantly increased amygdala activity during look anticipation (*p* = 0.003) in the low trait anxiety group (Figure 4D).

To explore the Treatment × Anticipation × Anxiety interaction in the bilateral posterior insula, parameter estimates from the clusters were subjected to separate three‐way mixed ANOVAs with the factors Treatment (OXT, PLC), Anxiety (high, low), and Anticipation (Look, Distance). In the left posterior insula, we observed a significant Treatment × Anxiety interaction (*F*[1, 61] = 5.883, *p* = 0.018, *η* ^2^_P_ = 0.088), and a significant Treatment × Anxiety × Anticipation interaction (*F*[1, 61] = 8.902, *p* = 0.004, *η* ^2^_P_ = 0.127) effect. Post hoc Bonferroni‐corrected analyses showed that OXT significantly attenuated left posterior insula activity during distance anticipation in the high trait anxiety group (*p* < 0.001). In the right posterior insula, we did not observe a main effect or interaction effects involving the factor Anxiety.

The whole‐brain full factorial ANOVA model with the factors Treatment (OXT, PLC) and Stimulus (LookNeu, LookNeg, DisNeg) did not identify any regions showing either a significant main effect of Treatment or a Treatment × Stimulus interaction effect during the stimulus presentation period, suggesting OXT may not modulate explicit cognitive emotion regulation during exposure to threatening stimuli.

#### 2.3.3 Associations of Anticipatory Amygdala Activity and Subsequent Behavioral Performance

**Figure 5:**
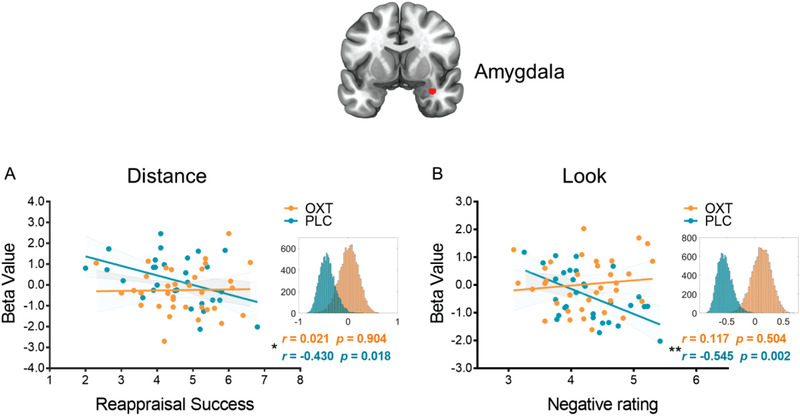
Associations of anticipatory amygdala (red cluster) activity and subsequent behavior performance. A) A significant negative correlation between anticipatory amygdala activity following the “Distance” cue and reappraisal success in the PLC group; B) a significant negative correlation between anticipatory amygdala activity following the “Look” cue and negative affect rating in the PLC group. Histograms indicate the bootstrapped distribution of correlation coefficients (10 000 iterations). * indicates the between‐group correlation differences. *p < 0.05; **p < 0.01; ***p < 0.001.

We next examined whether anticipatory amygdala activity was related to subsequent behavioral performance, and whether OXT modulated these associations. In general we only observed associations in the PLC group, such that anticipatory amygdala activity following the “Distance” cues showed a significant negative correlation with reappraisal success in the PLC but not in the OXT group (PLC: *r*[28] = −0.430, *p* = 0.018, 95% *CI*  = [−0.692; −0.102]; OXT: *r*[33] = 0.021, *p* = 0.904, 95% *CI* = [−0.374; 0.376]; Fisher's *z*: *z* = 1.84, *p* = 0.033, one tailed, *p* = 0.066, two tailed; **Figure** **5**A), anticipatory amygdala activity following the “Look” cues showed a significant negative correlation with the negative affect reported on the Look trials (LookNeg and LookNeu) in the PLC but not in the OXT group (PLC: *r*[28] = −0.545, *p* = 0.002, 95% *CI* = [−0.758; −0.260]; OXT: *r*[33] = 0.117, *p* = 0.504, 95% *CI* = [−0.235; 0.429]; Fisher's *z*: *z* = 2.79, *p* = 0.003, one tailed, *p* = 0.005, two tailed; Figure 5B). These results indicate that amygdala activity during the pre‐stimulus anticipation period was significantly associated with subsequent emotional reactivity and regulation following PLC treatment, and OXT uncoupled these associations.

#### 2.3.4 OXT Effects Were Moderated by Individual Differences in Trait Anxiety Levels and Emotion Regulation Ability

**Figure 6:**
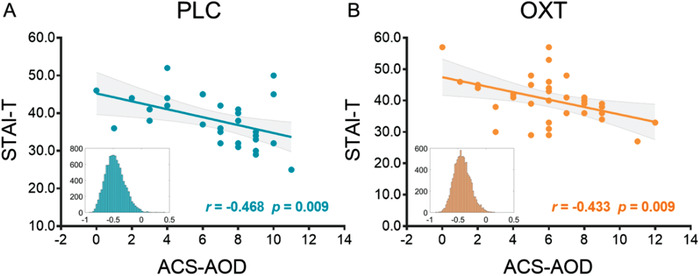
Correlation between STAI‐T and ACS‐AOD. A significant negative correlation between STAI‐T and ACS‐AOD in the A) PLC and B) OXT group. Histograms indicate the bootstrapped distribution of correlation coefficients (10 000 iterations).

To assess the trait anxiety levels and emotion regulation abilities, participants completed the trait subscale of the State‐Trait Anxiety Inventory (STAI‐T) and the prospective and decision‐related action orientation versus hesitation subscale of the Action Control Scale (ACS‐AOD) respectively. There were no significant pre‐treatment differences between treatment groups in trait anxiety (STAI‐T) and emotion regulation ability (ACS‐AOD; all *ps* > 0.05, see Table 1). First, we explored the association between STAI‐T and ACS‐AOD, and found a significant negative correlation between them both in the PLC (*r*[28] = −0.468, *p* = 0.009, 95% *CI* = [−0.724; ‐0.040]; **Figure** **6**A) and the OXT group (*r*[33] = −0.433, *p* = 0.009, 95% *CI* = [−0.706; −0.130]; Figure 6B).

**Figure 7:**
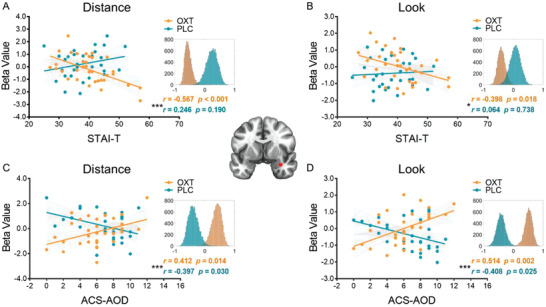
The mean parameter estimates from the amygdala (red cluster) during “Distance” anticipation were associated with A) STAI‐T and C) ACS‐AOD; The mean parameter estimates from the amygdala during the “Look” anticipation were associated with B) STAI‐T and D) ACS‐AOD. Histograms indicate the bootstrapped distribution of correlation coefficients (10 000 iterations). * indicates the between‐group correlation differences. *p < 0.05; **p < 0.01; ***p < 0.001.

In line with our hypotheses we next examined whether anticipatory amygdala activity was related to trait anxiety and emotion regulation ability. In contrast to the associations with behavioral indices, the scales exhibited significant associations in the OXT group: i) anticipatory amygdala activity following the “Distance” cue showed a significant negative correlation with STAI‐T in the OXT but not in the PLC group (PLC: *r*[28] = 0.246, *p* = 0.190, 95% *CI* = [−0.159; 0.559]; OXT: *r*[33] = −0.567, *p* < 0.001, 95% *CI* = [−0.752; −0.325]; Fisher's *z*: *z* = 3.42, *p* = 0.0003, one tailed, *p* = 0.0006, two tailed; **Figure** **7**A); ii) anticipatory amygdala activity following the “Look” cue showed a significant negative correlation with STAI‐T in the OXT but not in the PLC group (PLC: *r*[28] = 0.064, *p* = 0.738, 95% *CI* = [−0.311; 0.374]; OXT: *r*[33] = −0.398, *p* = 0.018, 95% *CI* = [−0.614; −0.125]; Fisher's *z*: *z* = 1.86, *p* = 0.031, one tailed, *p* = 0.063, two tailed; Figure 7B); iii) anticipatory amygdala activity following the “Distance” cue showed a significant positive correlation with ACS‐AOD in the OXT group but a significant negative correlation with ACS‐AOD in the PLC group (PLC: *r*[28] = −0.397, *p* = 0.030, 95% *CI* = [−0.690; −0.044]; OXT: *r*[33] = 0.412, *p* = 0.014, 95% *CI* = [0.091; 0.692]; Fisher's *z*: *z* = 3.28, *p* = 0.0005, one tailed, *p* = 0.001, two tailed; Figure 7C); iv) anticipatory amygdala activity following the “Look” cue showed a significant positive correlation with ACS‐AOD in the OXT group but a significant negative correlation with ACS‐AOD in the PLC group (PLC: *r*[28] = −0.408, *p* = 0.025, 95% *CI* = [−0.658; −0.085]; OXT: *r*[33] = 0.514, *p* = 0.002, 95% *CI* = [0.225; 0.732]; Fisher's *z*: *z* = 3.83, *p* = 0.0001, one tailed, *p* = 0.0001, two tailed; Figure 7D).

## 3 Discussion

The present pharmacological fMRI study examined the behavioral and neural effects of OXT on top‐down and bottom‐up emotion generation and regulation by means of a validated emotion regulation paradigm. Our results demonstrate that OXT affected neural activation during the anticipation but not the stimulus presentation phase. Specifically, we observed that OXT attenuated the activity in the bilateral posterior insular cortex and right amygdala during anticipation of top‐down regulation of a predictable threat stimulus (“Distance”), whereas it increased activation in the amygdala during bottom‐up anticipation of an unpredictable threat stimulus (“Look”). Moreover, the effects of OXT on emotion regulation success and anticipatory amygdala activity were modulated by individual differences in trait anxiety levels. OXT specifically attenuated amygdala activity during anticipation of top‐down regulation and subsequent emotion regulation success in participants with high trait anxiety, whereas it increased anticipatory amygdala activity for bottom‐up anticipation in participants with low trait anxiety. Together these findings support a regulatory role of OXT on anxiety‐related processing and further emphasize the context‐ and person‐dependent effects of intranasal OXT.^[^ ^55^, ^56^ ^]^

### 3.1 Top‐Down Guidance of Threat‐Related Perception and Attention

Previous studies indicate that top‐down and bottom‐up emotion generation and regulation processes rely on different neural systems and temporal dynamics.^[^ ^21^, ^22^ ^]^ Relative to stimulus‐driven bottom‐up processes, top‐down processes take into account cues or goals indicating upcoming threat‐related stimuli. These anticipatory cues or goals may contribute to perceptual and attentional prioritization of threatening stimuli.^[^ ^23^, ^24^ ^]^ To determine the top‐down and bottom‐up mechanisms during the present emotion regulation task, we extracted the time courses from hub regions of the visual and visuospatial attention networks and observed that top‐down anticipatory cues modulated the temporal dynamics of sensory coding and attention control. Specifically, top‐down cues accelerated the onset of stimulus coding in visual and visuospatial attention networks, suggesting that stimuli might be recognized more rapidly following top‐down (“Distance”) relative to bottom‐up (“Look”) cues.

### 3.2 Oxytocin Modulates Anticipatory Activation in the Posterior Insula and the Amygdala

With respect to OXT effects, we found that intranasal OXT attenuated amygdala and posterior insula activation during predictable top‐down aversive anticipation and that these effects were mainly driven by treatment effects in participants with high trait anxiety. Specifically, OXT increased activation in the amygdala during unpredictable bottom‐up aversive anticipation in low trait anxiety individuals. Several previous studies reported that OXT attenuated anxiety by reducing amygdala reactivity in response to threatening social stimuli,^[^ ^34^, ^36^, ^57^, ^58^ ^]^ which were primarily interpreted as anxiolytic effects of OXT. On the other hand, some studies showed that OXT does not reduce but rather increases threat responses in humans and animals in some contexts,^[^ ^39^, ^59^, ^60^ ^]^ suggesting that OXT may produce anxiolytic versus anxiogenic effects depending on the specific context as well as individual factors.^[^ ^55^, ^56^ ^]^ In contrast to a number of previous studies and our hypothesis, the present study did not reveal the effects of OXT on amygdala‐threat reactivity or emotion regulation during the stimulus presentation period. Attenuated amygdala threat reactivity following OXT has been repeatedly observed during the presentation of threatening social stimuli.^[^ ^34^, ^38^, ^61^, ^62^, ^63^ ^]^ Unlike previous studies, the present paradigm included a preceding cue that allowed participants to infer the probability of a subsequent threat‐stimulus and this cue might have shifted the effects of OXT to the anticipation period. The associations between amygdala activation during the anticipation period and level of induction of negative affect and regulation success observed in the PLC group further confirm OXT's impact on the anticipation period.

The amygdala plays a critical role in anxiety and fear as well as their regulation across different emotion regulation strategies,^[^ ^28^, ^29^, ^64^, ^65^ ^]^ with increasing evidence suggesting an important role in threat anticipation such that previous human and animal studies reported activation of the amygdala immediately before exposure to an anticipated imminent threat.^[^ ^66^, ^67^, ^68^ ^]^ Clinical studies have repeatedly reported increased amygdala activation during anticipation of aversive stimuli across anxiety disorders, including social anxiety disorder and generalized anxiety disorder.^[^ ^69^, ^70^ ^]^ The posterior insula/parietal operculum is strongly engaged in interoception, particularly the integration of interoceptive and exteroceptive multimodal sensory information, such as pain, touch, temperature, somato‐visceral sensations, auditory processing, and vestibular processing.^[^ ^71^, ^72^, ^73^, ^74^, ^75^ ^]^ Interoceptive information of the current physiological state of the body is progressively integrated with emotional awareness along a posterior to anterior gradient in the insula,^[^ ^73^ ^]^ and interoceptive signaling in the posterior/mid insula has been increasingly recognized as an important contributor to emotional experience including anxiety.^[^ ^76^ ^]^ Exaggerated anticipation of potential threat represents a key mechanism in anxiety disorders,^[^ ^77^ ^]^ and has been repeatedly associated with exaggerated amygdala and insula activation during the anticipation of a potential threat. For instance, Simmons et al. found that in anxiety‐prone individuals the anticipation of aversive visual stimuli was associated with increased activation in the insular cortex, including the posterior insula,^[^ ^78^ ^]^ and Tan et al. reported that posterior/middle insula interoception‐related activation was positively associated with trait and state anxiety levels.^[^ ^76^ ^]^ By using multifaceted anatomical and physiological circuit analyses in mice, Gehrlach et al. demonstrated a role for the posterior insula in the representation of anxiety‐related information and described an insula‐to‐central amygdala pathway which mediates anxiety‐related behaviors.^[^ ^79^ ^]^

These findings support a key role for the amygdala‐posterior insula circuit in anxiety‐related processing including threat anticipation.^[^ ^27^, ^80^, ^81^ ^]^ Several previous studies reported that OXT modulates amygdala reactivity during threat exposure with increasing evidence for modulatory effects on the posterior insula during anticipation of uncertain and potential painful stimulation,^[^ ^82^ ^]^ as well as on the functional connectivity of this region with the anterior insula during attentional switching from interoceptive towards external salient cues.^[^ ^83^ ^]^ In line with these previous findings, the current results emphasize that the anxiolytic or anxiogenic effects of OXT are neurally mediated by the insula‐amygdala circuit.

### 3.3 Oxytocin Differentially Modulated Amygdala Responses in High and Low Anxiety Individuals during Top‐Down (“Distance”) and Bottom‐Up (“Look”) Aversive Anticipation

Exploring the modulatory role of anxiety revealed that individual variations in pre‐treatment trait anxiety considerably affected the effects of OXT, such that it specifically attenuated amygdala and posterior insula activation during anticipation of top‐down regulation and subsequent emotion regulation success in the high anxiety group whereas it increased anticipatory activation in the amygdala for bottom‐up processing in the low anxiety group. The modulatory role of trait anxiety was further emphasized by correlational analyses that revealed that OXT induced a negative association between trait anxiety and anticipatory amygdala activation for both, top‐down and bottom‐up anticipation, with a visual inspection of the association indicating stronger OXT effects in high anxiety participants during top‐down anticipation, yet stronger effects in low anxiety participants during bottom‐up anticipation. These findings are in accordance with some prior results,^[^ ^51^, ^52^ ^]^ suggesting that trait anxiety may be an important modulator of the effects of oxytocin.

Substantial evidence indicates that high anxiety individuals overestimate threat value, which strengthens task‐irrelevant stimulus‐driven attention but impairs goal‐directed attention.^[^ ^20^, ^28^, ^84^ ^]^ The imbalance between bottom‐up and top‐down attention systems may result in emotion dysregulation, excessive anxiety, and threat‐related attentional bias.^[^ ^85^ ^]^ In contrast, low anxiety individuals may exhibit a lack of adequate arousal and vigilance to cope with the unexpected bottom‐up threat. In line with this conceptualization, individuals with higher trait anxiety demonstrate stronger attentional bias for threat‐related stimuli,^[^ ^86^ ^]^ allowing faster detection of potential threats in the environment, but deficient cognitive control of task‐irrelevant emotional stimuli.^[^ ^87^ ^]^ On the neural level individuals with high trait anxiety exhibit increased amygdala activity in response to,^[^ ^88^, ^89^ ^]^ as well as during the anticipation of threat stimuli,^[^ ^89^ ^]^ and faster escape decisions during threat anticipation in the context of the stronger amygdala and insula activation.^[^ ^54^ ^]^ This may represent one of the reasons why high anxiety individuals adaptively perform better than low anxiety individuals to identify threat stimuli under anxious conditions.^[^ ^90^ ^]^

Some evidence suggests that OXT attenuates attentional bias towards threat‐related stimuli,^[^ ^91^, ^92^ ^]^ indicating a potential role of OXT in attentional control and threat vigilance. In the present study, OXT may thus have facilitated top‐down goal‐directed attention by attenuating amygdala activity in high anxiety participants, while promoting bottom‐up attention/vigilance to unexpected threats by enhancing anticipatory amygdala activity in low anxiety participants. The opposite effects of OXT on anticipatory amygdala activation in high versus low anxiety individuals may suggest a baseline anxiety level dependent mechanism via which OXT promotes optimal levels of amygdala activation during the anticipation of an imminent threat. OXT may, therefore, be a potential intervention to promote an adaptive balance between bottom‐up and top‐down attention systems depending on individual levels of pre‐treatment trait anxiety levels. Given that some disorders are characterized by an imbalance between top‐down and bottom‐up threat processing, including anxiety disorders,^[^ ^84^ ^]^ schizophrenia,^[^ ^93^ ^]^ and autism,^[^ ^94^ ^]^ OXT may have a beneficial therapeutic effect in these disorders.

The cognitive processes of distancing reappraisal during the anticipation period mainly involve attentional control, whereas the stimulus presentation period additionally involves self‐projection, affective self‐reflection, and cognitive control.^[^ ^95^, ^96^, ^97^ ^]^ Combined with findings from our prior resting‐state study,^[^ ^98^ ^]^ it may be hypothesized that OXT has the potential to modulate pre‐stimulus attentional control of threatening information. Concerning the behavioral performance during the emotion regulation paradigm, we did not observe the effects of OXT per se, although the high anxiety group exhibited marginally significant improvement of reappraisal success following OXT, which may suggest that OXT has the potential to improve cognitive emotion regulation in high anxiety individuals. In addition, we hypothesized that OXT would increase PFC activity during distancing reappraisal, and thus may enhance explicit emotion regulation. However, we did not observe any effects of OXT on the PFC during emotion regulation. In accordance with prior findings, we observed that distancing reappraisal mainly recruited the default network.^[^ ^49^, ^97^ ^]^ The default network, comprising the precuneus/posterior cingulate cortex, anterior medial prefrontal cortex, medial temporal, and inferior parietal regions, plays a key role in internally oriented and self‐referential mental processes.^[^ ^99^, ^100^ ^]^ Our findings support the notion that distancing reappraisal involves internally‐oriented attention reflected by the recruitment of the default network. The primary engagement of the posterior regions in the default network by the current paradigm may have additionally contributed to the absence of OXT effects on the PFC in the present study.

### 3.4 Associations of Anticipatory Amygdala Activity and Subsequent Behavior Performance

Anticipation is a universal preparatory response to an upcoming or uncertain event and previous studies reported that pre‐stimulus anticipatory neural activity predicts subsequent emotion regulation success. For instance, pre‐stimulus functional connectivity between key areas of pain processing has been associated with pain susceptibility.^[^ ^101^ ^]^ Denny, Ochsner, et al. found that anticipatory brain activity predicts the success or failure of subsequent emotion regulation.^[^ ^32^ ^]^ In the present study, anticipatory amygdala activity was associated with subsequent emotion regulation performance only in the PLC group. Specifically, i) lower anticipatory amygdala activity after the “Distance” cue associated with higher reappraisal success, and, ii) higher anticipatory amygdala activity after the “Look” cue associated with lower negative affect ratings. In sum, decreased top‐down anticipatory amygdala activity was associated with improved explicit emotion regulation performance, whereas increased bottom‐up anticipatory amygdala activity was associated with lower induction of negative affect by the subsequent stimulus. The associations between pre‐stimulus amygdala activity and subsequent performance were not observed following OXT, indicating that OXT uncoupled the associations between anticipatory amygdala activation and subsequent emotional experience and emotion regulation.

### 3.5 Exploratory Analysis of Associations between Emotion Regulation Ability and Anticipatory Amygdala Activity Under OXT and PLC

We additionally explored associations between individual variations in emotion regulation ability (as assessed by the ACS‐AOD) and anticipatory amygdala activity. While no associations between anticipatory amygdala activity and trait anxiety levels were observed in the PLC group, we observed significant negative associations of anticipatory amygdala activity with emotion regulation ability in this group but significant positive associations in the OXT group. Visual inspection of the scatterplots suggests that OXT primarily decreased amygdala activity in individuals with a low emotion regulation ability during the top‐down (“Distance”) anticipatory period, whereas it increased amygdala activity in individuals with high emotion regulation ability during the bottom‐up (“Look”) anticipatory period. These findings indicate that the effects of OXT are not only modulated by “emotional reactivity” traits (anxiety) but also by emotion regulation relevant traits.

### 3.6 Cognitive Reappraisal from the Perspective of Large‐Scale Brain Networks

At the neural level, emotion regulation requires the concerted engagement of distributed brain systems beyond prefrontal regions such as dorsolateral PFC, dorsomedial PFC, and ventromedial PFC.^[^ ^29^, ^96^ ^]^ For instance, in the present paradigm, successful emotion regulation via distancing was accompanied by increased activation in multiple brain systems, such as the posterior default network, dorsal attention network, frontoparietal control network, and salience network (see Supporting Information). Research on interacting large‐scale networks that support successful emotion regulation may thus provide novel insights into the underlying neural mechanisms.^[^ ^102^, ^103^, ^104^, ^105^, ^106^ ^]^

## 4 Limitations and Conclusions

The present study focused on the effects of OXT in men, however, accumulating evidence from animal models suggests that the OXT system may have evolved a sexual dimorphic role in the domains of anxiety and stress regulation.^[^ ^107^, ^108^ ^]^ Initial studies that aimed at determining sexual dimorphic effects of intranasal OXT in humans reported opposite effects on amygdala activation in response to potentially threatening social stimuli,^[^ ^109^, ^110^ ^]^ and it remains to be determined whether the sexually dimorphic effects of OXT generalize to anticipatory amygdala threat activation. This study employed a cognitive emotion regulation paradigm to examine the modulatory effect of intranasal OXT on bottom‐up and top‐down emotion generation and regulation during the anticipation and presentation of threat stimuli. We found that OXT attenuated bilateral posterior insular cortex and right amygdala activation during anticipation of top‐down regulation of predictable threat stimuli in participants with high trait anxiety, whereas it increased activation in the amygdala during anticipation of bottom‐up unpredictable threat stimuli in participants with low trait anxiety. Collectively, these findings support a regulatory role of OXT on threat anticipation and further emphasize the context‐ and person‐dependent effects of intranasal OXT.

## 5 Experimental Section

##### Participants

*N* = 88 healthy, right‐handed male participants were enrolled in the study. To avoid confounding effects of hormonal changes across the menstrual cycle with OXT administration, the participants were males only. A total of 23 participants were excluded leading to a final sample size of *N* = 65 (randomized double‐blind allocation to 35 = OXT, 30 = PLC administration; mean age = 20.83 ± 2.60 years). The inclusion and exclusion criteria of the participants are provided in Supporting Information: Participants and CONSORT flow diagram (Figure S1, Supporting Information). To further explore the modulatory influence of trait anxiety on OXT effects, participants were assigned to “high” versus “low” trait anxiety groups based on median‐split of pre‐treatment scores on the STAI‐T^[^ ^111^ ^]^ (PLC, low anxiety: Mean ± SD  = 33.41 ± 3.45, high anxiety: Mean ± SD  = 44.38 ± 3.50; OXT, low anxiety: Mean ± SD = 34.19 ± 4.04, high anxiety: Mean ± SD = 45.32 ± 5.31).

The study was pre‐registered on the ClinicalTrials.gov database (Identifier: NCT03055546). All participants were free from current or past psychiatric, neurological, or other medical disorders (self‐report). Participants were instructed to abstain from alcohol and caffeine during the 24 h prior to the experiment. Written informed consent was obtained after a detailed explanation of the study protocol, study procedures had full ethical approval by the local ethics committee at the University of Science and Technology of China (Approval 276) and were in accordance with the latest revision of the Declaration of Helsinki.

##### Psychometric Assessment

Two days before MRI scanning, participants completed the Action Control Scale (ACS‐90) by Julius Kuhl (German version: HAKEMP 90, 1990).^[^ ^112^ ^]^ The ACS‐90 consists of three subscales: i) action orientation subsequent to failure versus preoccupation (AOF), ii) prospective and decision‐related action orientation versus hesitation (AOD), iii) action orientation during (successful) performance of activities (intrinsic orientation) versus volatility (AOP). Given that the AOD subscale has been previously associated with individual differences in 1) regulating negative emotions and stress,^[^ ^113^, ^114^ ^]^ 2) the effects of intranasal OXT on stress regulation,^[^ ^53^ ^]^ and 3) amygdala volume and intrinsic amygdala‐frontal functional connectivity,^[^ ^115^ ^]^ the examination of individual differences in pre‐treatment emotion regulation ability on the effects of OXT focused on this subscale. The Beck's Depression Inventory (BDI‐II)^[^ ^116^ ^]^ and the Autism Spectrum Quotient (AQ)^[^ ^117^ ^]^ were administered to control for pre‐treatment differences in the levels of depression and autism between treatment groups. Before drug administration and after MRI scanning, participants completed the Spielberger's State‐Trait Anxiety Inventory (STAI)^[^ ^111^ ^]^ and the Positive and Negative Affect Schedule (PANAS)^[^ ^118^ ^]^ to evaluate current emotional states. All participants were in the normal range with respect to depression, anxiety, and autism scores confirming the self‐reported lack of psychiatric conditions in the present sample.^[^ ^116^, ^117^ ^]^

##### Experimental Protocol

Figure 1A provides a schematic overview of the experimental protocol. In a double‐blind randomized PLC‐controlled between‐subject pharmaco‐fMRI design, participants self‐administered either a single dose of 24 international units (IU) OXT (ingredients: OXT, glycerin, sodium chloride, and purified water; Sichuan Meike Pharmaceutical Co. Ltd, Sichuan, China) or PLC nasal spray (produced by Sichuan Meike Pharmaceutical Co. with identical ingredients except for OXT) corresponding to three puffs per nostril. Based on previous pharmaco‐kinetic experiments,^[^ ^119^ ^]^ treatment was administered 35–40 min before the acquisition of the functional MRI time‐series.

The subsequent MRI acquisition included a 7.5 min resting‐state scan (Rest), four implicit emotion regulation task runs lasting 4 min 50 s each (emotional Go/NoGo), and six explicit emotion regulation task runs lasting 6 min 46 s each (distancing reappraisal) followed by a surprise memory paradigm outside of the scanner. Results from the implicit emotion regulation and memory paradigm will be reported in separate publications. During the resting‐state fMRI acquisition, participants were instructed to passively view a fixation cross and keep as motionless as possible. The brain structural data was acquired between the emotional Go/NoGo task and distancing reappraisal task so that there was a cognitive flushing period of 5 min between the tasks (washout). Head movements were minimized by using comfortable head cushions. In a paper‐pencil rating after MRI scanning, the participants answered two questions to evaluate their performance in the reappraisal task: i) indicate how successful you were in regulating your emotion on a scale from 1 (not successful at all) to 7 (very successful), ii) can you vividly imagine the scene/situation depicted in the pictures?

##### Stimuli and Reappraisal Task Design

70 min after drug administration, participants underwent a distancing reappraisal paradigm with the concomitant fMRI acquisition. The design of the task is shown in Figure 1B and represents a modified version of prior evaluated event‐related cognitive reappraisal paradigms.^[^ ^47^, ^49^, ^120^ ^]^ Each trial began with a 2 s cue (“Distance” or “Look”), followed by a 1.5–3.5 s anticipatory interval during which a fixation cross was presented. The image was presented for 8 s (threatening or neutral) in a pseudo‐random order across participants. Following the stimulus, a fixation cross was presented for a 2–5 s jittered interval followed by rating for 4 s during which participants were asked to rate how negative they felt on a scale of 1–9 (1 = not at all negative, 5 = medium negative, 9 = very negative). The rating was followed by a 4–7 s jittered intertrial interval (ITI).

The task included three types of trials: Look Neutral (LookNeu), Look Negative (LookNeg), and Distance Negative (DisNeg). For the “Look” condition, participants were instructed to simply view the image, understand its content, and react naturally without trying to regulate their emotions in any way. For the “Distance” condition, they were instructed to employ the distancing technique in order to view the stimulus from the perspective of a detached and objective observer.^[^ ^47^, ^97^ ^]^ The “Distance” cues provided participants a top‐down goal to regulate their negative emotion, whereas the “Look” cues simply required participants’ bottom‐up emotional response to the subsequent images. Participants were specifically instructed not to close their eyes or look away from the images. The formal experiment did not start until the experimenters confirmed that the participant had fully understood and mastered the distancing reappraisal strategy during a pre‐scanning training.

Stimuli for the reappraisal task incorporated 30 neutral and 60 aversive pictures from the International Affective Picture System (IAPS, National Institute of Mental Health Center for Emotion and Attention, University of Florida) database.^[^ ^121^ ^]^ Since OXT's effects were documented as being more pronounced for social stimuli than non‐social stimuli, all stimuli depicted threatening social scenes (e.g., corpses, bodily mutilation or assaults) and neutral social situations (e.g., shopping, at work). In a pre‐study, the pictures were initially rated by an independent sample (*n* = 37; 18 males) on 9‐point scales for the following dimensions: valence (ranging from 1 for unpleasant to 9 for pleasant), arousal (ranging from 1 for calm to 9 for aroused). Participants completed 6 functional runs, each of which contained 15 trials. The last two runs were removed from analyses since participants generally self‐reported increased fatigue during the reappraisal task. To objectively confirm across‐run fatigue effects,^[^ ^122^ ^]^ the parameter estimates were extracted during stimuli presentation from the regions involved in visual expertise, including higher visual network, primary visual network, and fusiform gyrus. Results revealed reduced activation in these visual regions across the six runs (Figure S2, Supporting Information) confirming self‐reported increasing fatigue over time. Therefore, the final stimuli for all analyses included 20 neutral (“LookNeu”: valence = 5.33 ± 0.37, arousal = 3.32 ± 0.32; mean ± SD) and 40 negative (20 “LookNeg”: valence = 2.33 ± 0.50, arousal = 6.96 ± 0.73; 20 “DisNeg”: valence = 2.34 ± 0.54, arousal = 6.96 ± 0.77; mean ± SD) pictures. The valence and arousal of negative pictures were matched between “DisNeg” and “LookNeg” conditions (valence: *P* = 0.995, arousal: *P* = 0.980, two‐sample t‐tests).

##### Behavioral Analyses

To identify the main and interaction effects of treatment and trait anxiety on affect rating, the affect rating for each participant was subjected to a mixed‐effect ANOVA, with two between‐subject factors Treatment (OXT, PLC) and Anxiety (high, low) and a within‐subject factor Condition (LookNeu, LookNeg, DisNeg). The interaction effect of treatment and trait anxiety on reappraisal success was examined using a univariate ANOVA with factors treatment (OXT, PLC) and anxiety (high, low). Reappraisal success was defined as the decrease in reported negative affect rating on DisNeg trials versus LookNeg trials (i.e., LookNeg − DisNeg value) for each participant.^[^ ^120^, ^123^ ^]^ Post‐hoc pairwise comparisons were corrected for multiple comparisons with Bonferroni corrections. Corresponding analyses were employed using SPSS (version 22.0; IBM SPSS Statistics, Armonk, NY, USA).

##### fMRI Data Acquisition

MRI data were collected on a 3T Siemens Trio scanner (Siemens Magnetom Trio TIM, Erlangen, Germany). High‐resolution brain structural data were acquired using a T1‐weighted sequence (repetition time [TR] = 1900 ms, echo time [TE] = 2.5 ms, flip angle = 9°, thickness = 1 mm with no gap, field of view [FOV] = 256 mm × 256 mm, image matrix = 256 × 256, 176 sagittal slices, voxel size = 1 × 1 × 1 mm) to improve spatial normalization of the functional data. Functional data were acquired using a T2*‐weighted Echo Planar Imaging (EPI) sequence with the following parameters: TR/TE = 2000/30 ms, flip angle = 90°, acquisition matrix = 64 × 64, voxel size = 3.1 × 3.1 × 3 mm, FOV = 200 × 200 mm^2^, transverse slices = 33, thickness/gap = 3/0.6 mm.

##### fMRI Data Analysis

fMRI images were preprocessed and analyzed using the standard procedure in SPM12 (http://www.fil.ion.ucl.ac.uk/spm/; Wellcome Trust Centre for Neuroimaging) and DPABI (Version: V4.2_190 919; http://rfmri.org/dpabi).^[^ ^124^ ^]^ The first five volumes of each functional run were removed to allow for MRI signal equilibrium. The preprocessing steps included slice timing, head motion correction, spatial normalization, and smoothing (8 mm full‐width at half maximum [FWHM] Gaussian kernel). For each participant and each run, a general linear model (GLM) was constructed by convolving the regressors with the canonical hemodynamic response function (HRF) to estimate evoked blood‐oxygen‐level‐dependent (BOLD) activity. The GLM contained regressors indicating the cue (duration = 0 s), anticipation (duration = 1.5–3.5 s, subject‐specific time), stimulus (duration = 8 s), rating phase (duration = 4 s), and six motion‐correction parameters.

It has been demonstrated that top‐down expectations can improve stimulus detection and coding. Expected stimuli can be recognized more rapidly than unexpected ones.^[^ ^20^, ^23^, ^125^, ^126^ ^]^ Top‐down modulation of visual processing involves visual cortical areas that are involved with object recognition, as well as the parietal and prefrontal cortex which are involved with visually guided movements and attentional control.^[^ ^127^ ^]^ To manifest the mechanism of top‐down and bottom‐up processes in the distancing reappraisal paradigm, the time courses were extracted from the regions of interest (ROIs) in the primary visual network, higher visual network, fusiform gyrus, and visuospatial attention network during stimuli presentation period. The ROIs of visual and visuospatial attention networks were created based on the Stanford Resting‐State Network templates (http://findlab.stanford.edu/functional_ROIs.html).^[^ ^128^ ^]^ The fusiform gyrus mask was created using the Automated Anatomic Labelling (AAL) atlas,^[^ ^129^ ^]^ implemented in WFU Pickatlas.^[^ ^130^ ^]^ The anatomical location and Brodmann areas of each ROI in the primary visual, higher visual, fusiform gyrus, and visuospatial attention networks are summarized in Table S1, Supporting Information.

To determine the effects of OXT on the anticipation of threat, an ANOVA model was conducted by entering contrast estimates obtained from first‐level models into a full factorial model with factors Treatment (OXT, PLC) and Anticipation (Look, Distance). A separate ANOVA was conducted to examine the effects of OXT on the stimulus presentation phase with factors Treatment (OXT, PLC) and Stimulus type (LookNeu, LookNeg, DisNeg). Results were reported when passing a whole‐brain FWE‐corrected extent threshold of *p* < 0.05 at the cluster level or small‐volume correction at the FWE‐corrected threshold of *p* < 0.05 within the anatomically defined bilateral amygdala. The amygdala was chosen based on previous studies demonstrating OXT‐induced attenuated amygdala threat reactivity.^[^ ^36^, ^39^ ^]^ The amygdala mask was created using the AAL atlas,^[^ ^129^ ^]^ implemented in WFU Pickatlas.^[^ ^130^ ^]^ fMRI mean contrast values for subsequent analyses were extracted from a 4‐mm radius sphere centered at the peak coordinate of the amygdala cluster.

##### Associations between Neural and Behavioral Data

To explore associations between amygdala activity and behavior, Pearson correlations were calculated between the mean beta parameter estimates from the amygdala cluster and behavioral performance (i.e., reappraisal success) across participants. Furthermore, the modulatory influences of individual variations in anxiety (trait anxiety as a measure of bottom‐up reactivity) and emotion regulation ability (ACS‐AOD as a measure of top‐down regulatory control) on OXT effects were examined. To this end, nonparametric bootstrap tests (10000 iterations) were conducted on a correlation coefficient to obtain a 95% confidence interval (CI) estimate of Pearson's r. Between‐group correlation differences were tested using Fisher's *z*‐tests. Based on findings from previous studies reporting stronger OXT effects in individuals with higher trait anxiety,^[^ ^51^, ^52^ ^]^ between‐group correlation differences were tested one‐tailed.

## Conflict of Interest

The authors declare no conflict of interest.

## Author Contributions

F.X. and B.B. designed the study; data collection was done by F.X., X.Z., Z.Z., X.Y., Q.W., and Y.G.; F.X., X.Z., and D.D. performed data analysis; interpretation of data and drafting of the manuscript was done by F.X., B.B., A.C., and K.M.K.

## Supporting information

Supporting Information
