# Supplementary material for: Oxytocin Differentially Modulates Amygdala Responses during Top‐Down and Bottom‐Up Aversive Anticipation
Source: Adv Sci (Weinh). 2020 Jul 1;7(16):2001077. doi: 10.1002/advs.202001077 (PMC7435249; doi:10.1002/advs.202001077)
Supplement: Supplementary file 1 — Supporting Information [file ADVS-7-2001077-s001.pdf]

Supporting Information

Oxytocin Differentially Modulates Amygdala Responses during Top-Down and Bottom-Up Aversive Anticipation

Authors

Fei Xin, Xinqi Zhou, Debo Dong, Zhongbo Zhao, Xi Yang, Qianqian Wang, Yan Gu, Keith M. Kendrick, Antao Chen, Benjamin Becker**

Participants

N = 88 healthy, right-handed male participants were enrolled in the study. A total of 23 participants were excluded leading to a final sample size of N = 65. Three participants were excluded due to incomplete data. The present study involved emotion perception and emotion regulation, which has been demonstrated to be influenced by individuals' depressive and autistic traits.[1,2] Four participants with high depressive and autistic traits were therefore excluded from all subsequent analyses (BDI-II > 28; AQ > 30, in ref. [3]). To match the trait anxiety scores between OXT and PLC groups, three participants with high trait anxiety (STAI-T > 60) in the OXT group were excluded. Four participants were excluded due to excessive head motion (>3 mm translation, >3° rotation). One participant whose mean negative affect rating of reappraisal stimuli (i.e. DisNeg) was beyond three standard deviations had to be excluded. Six participants whose mean negative affect ratings of neutral stimuli (i.e. LookNeu) were larger than 3 had to be excluded. Two participants whose accuracy during a surprise memory test were beyond three standard deviations had to be excluded. See **Figure S1** for the respective CONSORT flow diagram.

CONSORT
TRANSPARENT REPORTING of TRIALS

CONSORT 2010 Flow Diagram

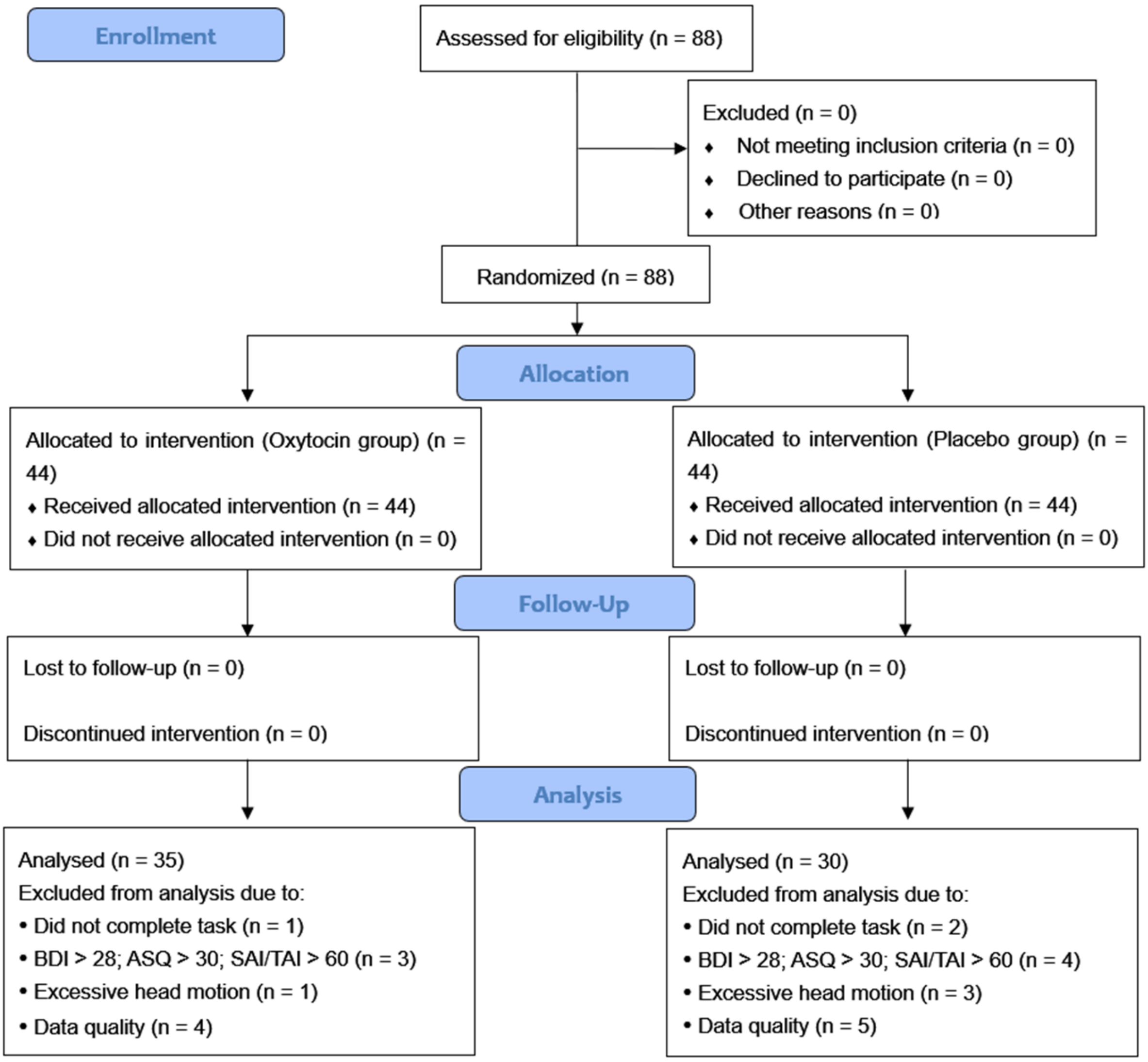

Figure S1. The CONSORT flow diagram.

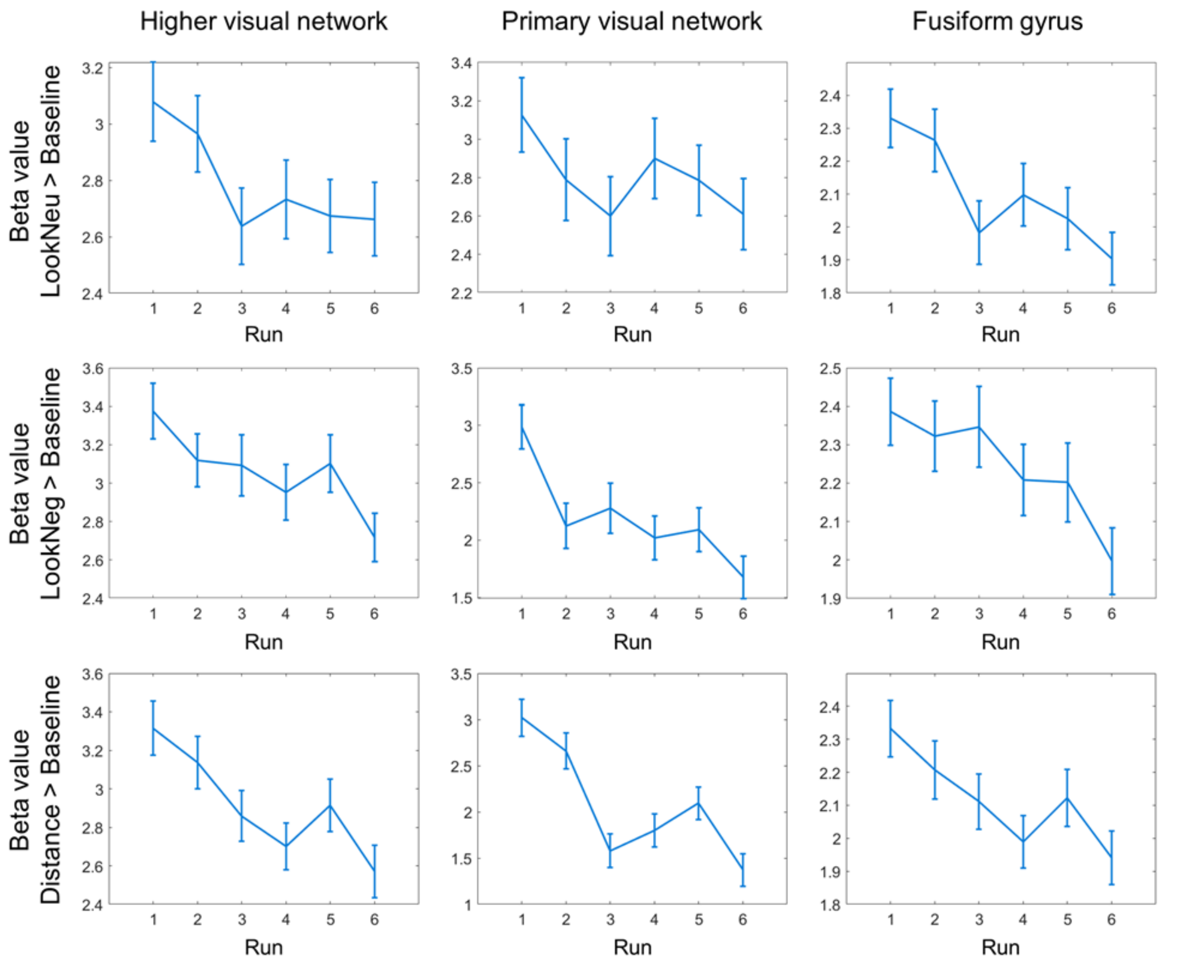

Figure S2. Across-run fatigue. Reduced activation in the higher visual network, primary visual network, and fusiform gyrus during the stimulus presentation across the six runs.

Table S1. Anatomical location and Brodmann areas of each ROI in the primary visual network, higher visual network, fusiform gyrus, and visuospatial attention network.

Network	Anatomical Location of Functional ROIs	Brodmann Areas
Primary Visual Network	Calcarine Sulcus	17
Higher Visual Network	Left Middle Occipital Gyrus, Superior Occipital Gyrus	18, 19, 17
	Right Middle Occipital Gyrus, Superior Occipital Gyrus	17, 18, 19
Visuospatial Attention Network	Left Middle Frontal Gyrus, Superior Frontal Cortex, Precentral Gyrus	6
	Left Inferior Parietal Sulcus	2, 40, 7
	Left Frontal Operculum, Inferior Frontal Gyrus	44, 48, 45
	Left Inferior Temporal Gyrus	37
	Right Middle Frontal Gyrus	6
	Right Inferior Parietal Lobule	2, 40, 7
	Right Frontal Operculum, Inferior Frontal Gyrus	44, 48
	Right Middle Temporal Gyrus	37
Fusiform Gyrus	Left Fusiform	37
	Right Fusiform	37

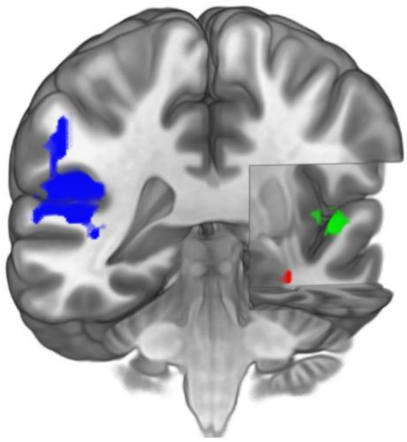

Figure S3. A significant Treatment × Anticipation interaction was observed in the posterior insula (left: [-48, -36, 18], blue; right: [48, -9, 12], green) and amygdala (right: [33, 0, -24], red).

General activation patterns engaged during the paradigm

Examining the DisNeg vs. LookNeg contrast during stimulus presentation revealed widespread activity in the posterior default network and frontoparietal control network (see **Figure S4**A and **Table S2**). Examination of the LookNeg vs. DisNeg contrast during stimulus presentation revealed activity in the visual network, fusiform gyrus, and regions within the dorsal attention network (see **Figure S4B** and **Table S3**). Examination of the DisNeg vs. LookNeg contrast during the pre-stimulus anticipation period revealed activity in the posterior default network and regions within the frontoparietal control network (see **Figure S6** and **Table S4**). Analysis of the the LookNeg vs. DisNeg contrast during the pre-stimulus anticipation period revealed no significant differences. In general these results resemble previous findings, [4,5] and confirm that our distancing reappraisal paradigm engaged the previously reported emotion regulation networks. We additionally extracted the time courses from the clusters in the visual and attention networks in the LookNeg vs. DisNeg contrast during the stimulus presentation. Time courses confirmed that the 'Distance' cue accelerated the onset of stimulus coding, suggesting that the visual and attention networks are engaged more rapidly after top-down 'Distance' cues than bottom-up 'Look' cues (see **Figure S5**).

Table S2. DisNeg vs. LookNeg contrast during the stimulus presentation.

Region	H.	Abbr.	MNI-coordinates			Cluster Size	Peak *T*
			x	y	z		
Stimulus presentation: DisNeg-LookNeg (OXT+PLC)							
Precuneus	L	PCu	-9	-57	33	832	11.677
Cuneus	R	Cun	9	-90	21		8.428
Cuneus	L	Cun	-3	-93	15		7.902
Angular gyrus	L	AnG	-45	-66	39	515	9.863
Angular gyrus	L	AnG	-48	-63	30		9.572
Superior temporal gyrus	R	STG	69	-36	3	152	6.974
Middle temporal gyrus	R	MTG	51	-33	0		6.638
Middle temporal gyrus	L	MTG	-63	-36	-3	99	6.964
Middle frontal gyrus	L	MFG	-39	6	54	88	6.181
Middle frontal gyrus	L	MFG	-39	15	45		5.525
Middle temporal gyrus	L	MTG	-54	-3	-24	78	5.978
Middle temporal gyrus	L	MTG	-60	-18	-21		5.239
Superior frontal gyrus	L	SFG	-12	18	63	10	5.473
Middle cingulate gyrus	L	MCgG	0	-21	36	3	5.138
Superior frontal gyrus	R	SFG	15	18	60	3	5.126
Middle temporal gyrus	R	MTG	66	-9	-24	3	5.091
Superior frontal gyrus	L	SFG	-15	51	33	2	4.985

H, hemisphere; MNI, Montreal Neurological Institute; L, left; R, right. Whole-brain FWE corrected at peak-level, $P < 0.05$.

Table S3. LookNeg vs. DisNeg contrast during stimuli presentation.

Region	H.	Abbr.	MNI-coordinates x	y	z	Cluster Size	Peak *T*
Stimuli presentation：LookNeg – DisNeg (OXT+PLC)							
Lingual gyrus	R	LiG	6	-66	3	1011	10.505
Fusiform gyrus	R	FuG	30	-45	-9		10.179
Lingual gyrus	L	LiG	-3	-66	3		9.918
Middle occipital gyrus	L	MOG	-33	-84	24	172	9.858
Fusiform gyrus	L	FuG	-27	-45	-12	532	9.005
Inferior occipital gyrus	L	IOG	-24	-93	-6		8.117
Fusiform gyrus	L	FuG	-30	-60	-9		7.943
Postcentral gyrus	L	PoG	-45	-30	48	633	8.589
Postcentral gyrus	L	PoG	-54	-24	27		8.409
Precentral gyrus	L	PrG	-39	-18	63		7.965
Middle occipital gyrus	R	MOG	39	-78	30	375	8.109
Superior parietal lobule	R	SPL	30	-63	42		6.290
Superior parietal lobule	R	SPL	30	-54	48		5.874
Supramarginal gyrus	R	SMG	51	-27	45	222	6.708
Supramarginal gyrus	R	SMG	60	-18	30		5.724
Precentral gyrus	L	PrG	-48	3	30	41	6.315
Precentral gyrus	R	PrG	51	9	30	44	6.259
Inferior temporal gyrus	R	ITG	54	-48	-12	42	6.251
Amygdala	R	AmG	21	0	-18	11	5.834
Superior parietal lobule	L	SPL	-21	-63	45	31	5.798
Posterior insula	L	PIns	-36	-3	-9	18	5.549
Middle frontal gyrus	R	MFG	27	3	57	2	5.002
Precuneus	R	PCu	24	-57	18	1	4.965

H, hemisphere; MNI, Montreal Neurological Institute; L, left; R, right. Whole-brain FWE corrected at peak-level, $P < 0.05$.

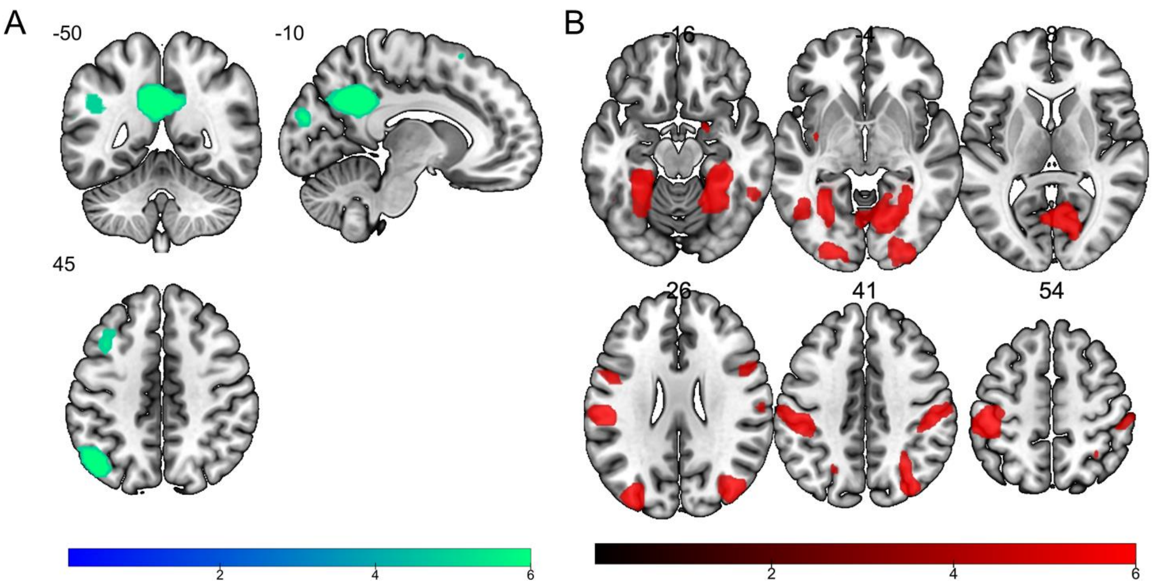

Figure S4. A) DisNeg vs. LookNeg contrast and B) LookNeg vs. DisNeg contrast during the stimuli presentation period.

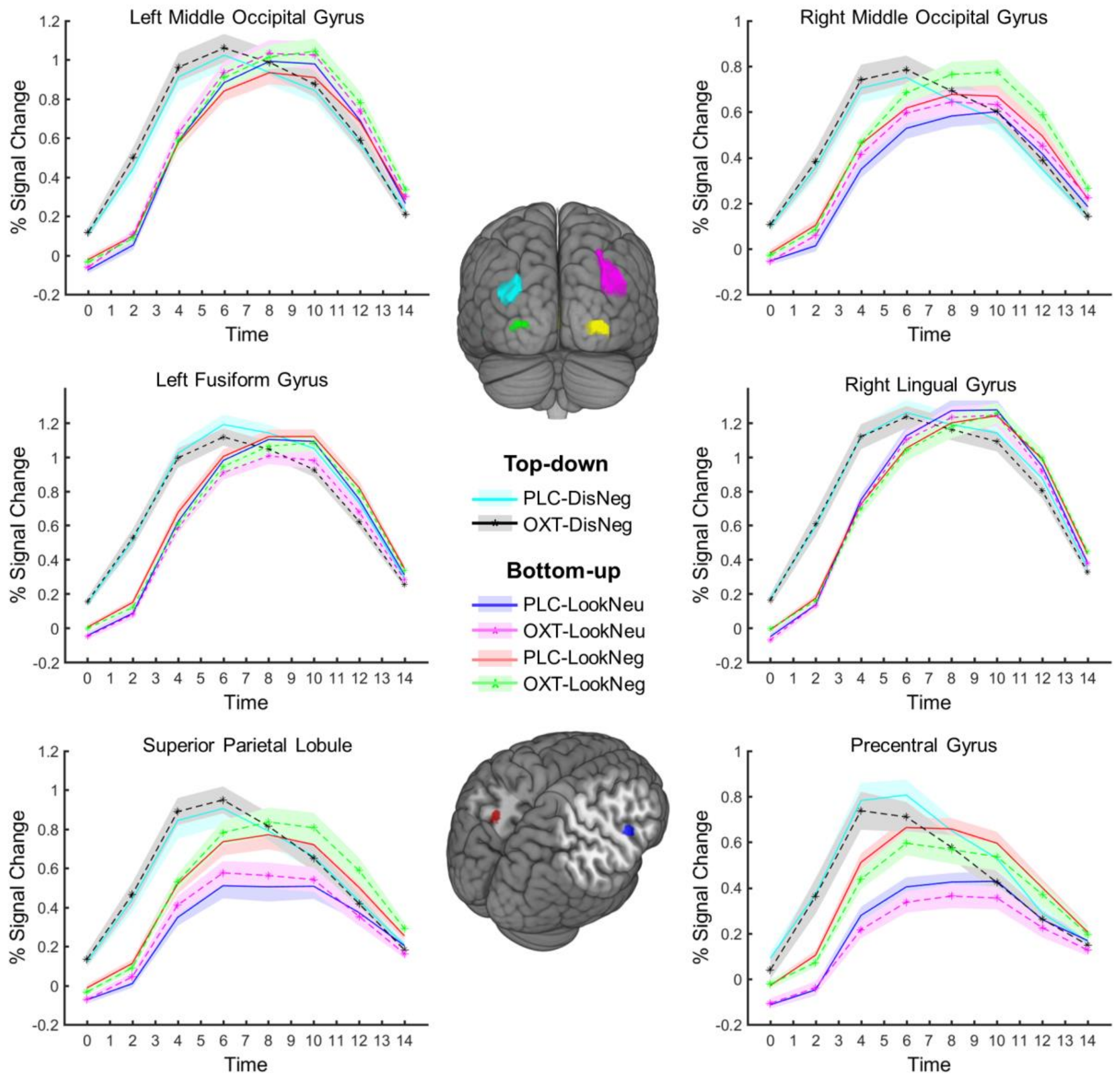

Figure S5. Top-down cues (i.e. 'Distance') accelerated stimulus coding in visual perception and sensory regions. Time courses reflect that the visual and attention networks were activated more rapidly after the 'Distance' than the 'Look' cue. Time courses were extracted from the clusters in the LookNeg vs. DisNeg contrast during stimulus presentation. All regions survived peak-level FWE correction, $P < 0.05$, see **Table S3**. Cyan: Left middle occipital gyrus, [-33, -84, 24]; Violet: Right Middle Occipital Gyrus, [39, -78, 30]; Green: Left fusiform gyrus, [-27, -45, -12]; Yellow: Right lingual gyrus, [6, -66, 3]; Red: Superior Parietal Lobule, [-21, -63, 45]; Blue: Precentral Gyrus, [51, 9, 30].

Table S4. DisNeg vs. LookNeg contrast during the pre-stimulus anticipation.

Region	H.	Abbr.	MNI-coordinates x	y	z	Cluster Size	Peak *T*
Pre-stimulus anticipation: DisNeg - LookNeg (OXT+PLC)							
Precentral gyrus	L	PrG	-51	6	39	135	7.660
Angular gyrus	L	AnG	-45	-63	15	296	7.405
Occipital fusiform gyrus	L	OFuG	-36	-72	-12		6.768
Middle temporal gyrus	L	MTG	-57	-45	-12		6.308
Angular gyrus	L	AnG	-39	-66	42	233	7.226
Angular gyrus	L	AnG	-39	-54	45		6.241
Inferior occipital gyrus	R	IOG	42	-75	9	116	7.011
Middle occipital gyrus	R	MOG	33	-78	18		6.495
Precuneus	L	PCu	-3	-75	39	300	6.763
Precuneus	L	PCu	-3	-66	36		6.185
Calcarine cortex	L	Calc	-12	-72	9		6.096
Middle cingulate gyrus	L	MCgG	-6	-24	42	48	6.726
Posterior cingulate gyrus	L	PCgG	-3	-33	36		5.527
Occipital fusiform gyrus	R	OFuG	36	-63	-12	235	6.581
Occipital fusiform gyrus	R	OFuG	30	-75	-12		6.381
Occipital fusiform gyrus	R	OFuG	30	-63	-6		6.310
Supramarginal gyrus	R	SMG	51	-27	42	120	6.454
Superior parietal lobule	R	SPL	39	-45	57		5.754
Supramarginal gyrus	R	SMG	60	-21	27		5.488
Precentral gyrus	R	PrG	48	9	27	124	6.395
Precentral gyrus	R	PrG	51	9	39		6.268
Middle frontal gyrus	L	MFG	-33	3	66	66	6.304
Middle frontal gyrus	L	MFG	-39	9	57		5.871
Thalamus proper	L	TP	-3	-15	12	15	5.786
Posterior cingulate gyrus	L	PCgG	-6	-45	9	13	5.737
Precentral gyrus	R	PrG	45	-6	54	18	5.592
Superior frontal gyrus	L	SFG	-15	12	63	7	5.578
Triangular part of the inferior frontal gyrus	L	TrIFG	-48	36	3	6	5.453
Precentral gyrus	L	PrG	-24	-21	72	4	5.360
Superior frontal gyrus	L	SFG	-15	6	72	2	5.268
Precentral gyrus	R	PrG	39	-21	69	3	5.245
Lingual gyrus	L	LiG	-3	-75	-3	5	5.189
Posterior cingulate gyrus	R	PCgG	6	-42	0	8	5.150
Posterior cingulate gyrus	R	PCgG	9	-42	9		5.149
Superior frontal gyrus	L	SFG	-27	15	63	3	5.115

H, hemisphere; MNI, Montreal Neurological Institute; L, left; R, right. Whole-brain FWE corrected at peak-level, $P < 0.05$.

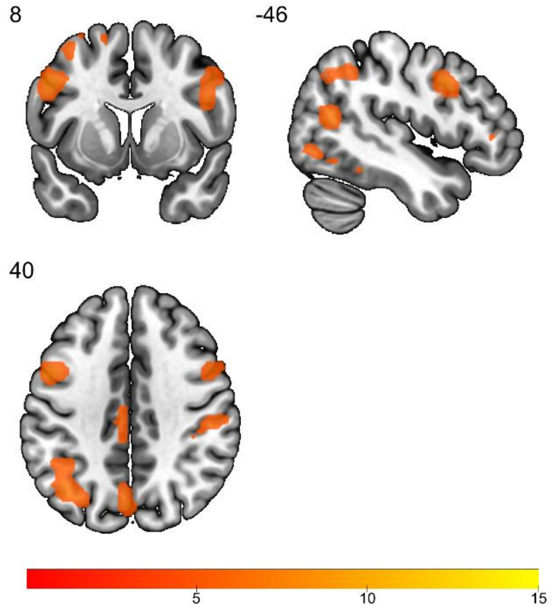

Figure S6. DisNeg vs. LookNeg contrast during the pre-stimulus anticipation.

References

[1] E. Poljac, E. Poljac, J. Wagemans, *Autism* **2013**, *17*, 668-680.

[2] M. M. Rive, G. van Rooijen, D. J. Veltman, M. L. Phillips, A. H. Schene, H. G. Ruhé, *Neuroscience & Biobehavioral Reviews* **2013**, *37*, 2529-2553.

[3] M. Liu, *Bulletin of Special Education* **2008**, *33*, 73-92.

[4] H. W. Koenigsberg, J. Fan, K. N. Ochsner, X. Liu, K. G. Guise, S. Pizzarello, C. Dorantes, S. Guerreri, L. Tecuta, M. Goodman, A. New, L. J. Siever, *Biological Psychiatry* **2009**, *66*, 854-863.

[5] X. Xie, S. Mulej Bratec, G. Schmid, C. Meng, A. Doll, A. Wohlschlager, K. Finke, H. Forstl, C. Zimmer, R. Pekrun, L. Schilbach, V. Riedl, C. Sorg, *NeuroImage* **2016**, *134*, 270-280.
